# Synergetic role of inhibition and excitation in bursting synchronization

**DOI:** 10.1186/1471-2202-13-S1-P179

**Published:** 2012-07-16

**Authors:** Igor Belykh

**Affiliations:** 1 Department of Mathematics and Statistics and Neuroscience Institute, Georgia State University, 30 Pryor Street, Atlanta, GA 30303, USA

## 

We study the influence of coupling strength and network topology on synchronization in strongly coupled networks of square-wave bursters with fast excitatory and inhibitory connections. Fast excitation is known to promote synchrony, which appears in purely excitatory networks of bursting neurons as long as the excitatory coupling exceeds a threshold value. On the contrary, fast strong inhibition leads to anti-phase or asynchronous bursting in purely inhibitory networks.

In this work, we report a surprising find that the addition of strong fast inhibitory connections to excitatory networks of square-wave bursters can promote synchrony. More precisely, the inhibitory connections, introduced to an excitatory network, can lower the synchronization threshold much more significantly than strengthening the present excitatory connections.

We use the stability methods, developed in [1,2], to explain this synergetic role of otherwise destabilizing inhibition in promoting stable synchronization of bursting neurons in excitatory networks. We demonstrate that there is a balance between the excitatory and inhibitory couplings providing the maximum stability of synchronization. These results are applicable to synchronization in a pair of mutually connected neurons as well as to large networks with mixed excitatory-inhibitory couplings. We also study the interplay between burst synchronization and the excitatory and inhibitory networks’ structures and show that the number of excitatory and inhibitory inputs each neuron receives is often the crucial quantity.

## References

[B1] Belykh I de Lange E Hasler M Synchronization of bursting neurons: what matters in the network topology Phys Rev Lett 2005 94 188101 15904412 10.1103/PhysRevLett.94.188101

[B2] Belykh I Hasler M Mesoscale and clusters of synchrony in networks of bursting neurons Chaos 2011 21 016106 10.1063/1.3563581 21456848

